# Longitudinal Physical Development of Future Professional Male Soccer Players: Implications for Talent Identification and Development?

**DOI:** 10.3389/fspor.2020.578203

**Published:** 2020-10-21

**Authors:** Chris Saward, Mark Hulse, John G. Morris, Heita Goto, Caroline Sunderland, Mary E. Nevill

**Affiliations:** ^1^Department of Sport Science, Sport, Health and Performance Enhancement (SHAPE) Research Centre, School of Science and Technology, Nottingham Trent University, Nottingham, United Kingdom; ^2^Manchester United Football Club, AON Training Complex, Manchester, United Kingdom; ^3^Faculty of Sports Science, Kyushu Kyoritsu University, Fukuoka, Japan

**Keywords:** association football, stature, body mass, agility, sprint speed, counter-movement jump, endurance, field-tests

## Abstract

The present study examined if elite youth male association football (soccer) players aged 8–19 y (*n* = 2,875) from the English talent development system, who ultimately achieved professional status differed in stature, body mass, and physical performance (20-m sprint speed, slalom agility speed, vertical counter-movement jump with arm swing jump height, multistage fitness test distance) compared with their non-professional peers. The study also examined the longitudinal pattern of development of stature, body mass, and physical performance, and if this was different between future professionals and non-professionals, while considering the effects of playing position. Multilevel modeling of the 8,898 individual (player-occasion) data points suggested that from age 12.0, the future professionals performed better in a vertical counter-movement jump with arm swing test and slalom agility test than future non-professionals, and improved at a faster rate, so that by age 18.0 the differences in vertical counter-movement jump with arm swing and slalom agility performance were 1.7 cm (*p* < 0.001, *d* = 0.3) and 0.14 s (*p* < 0.001, *d* = 0.5), respectively. In addition, future professionals were faster (by 0.02–0.04 s on the 20-m sprint, *p* < 0.001, *d* = 0.2) and ran further in the multistage fitness test (by 47 m, *p* = 0.014, *d* = 0.2) than future non-professionals throughout their development, but there were no differences in stature or body mass during development between the groups. Whereas, multistage fitness test performance improved linearly with age, the development of all other physical characteristics was non-linear. There were inter-individual differences in the development of all characteristics, and there were differences between playing positions in the development of all characteristics. Thus, in summary, future professionals jump higher, are more agile, faster, and more endurance fit than future non-professionals as they age, and the pattern of development is different in professionals and non-professionals for vertical jumping and slalom agility performance.

## Introduction

Talent identification and development in association football (soccer) refers to the interlinked and ongoing processes of: recognizing young players with the potential to become elite senior players; and providing them with the most appropriate learning environment to realize their potential (Williams and Reilly, 2000). In England, elite male youth players are exposed to a talent identification and development process which is largely based on scouting and recruitment to academies affiliated with professional clubs. Once recruited, academies attempt to provide players with a systematic programme of coaching and support, and make ongoing judgements of players' potential to succeed. The nature of this academy system is governed by a recent strategic framework, The Elite Player Performance Plan (EPPP), which aims to increase the number and quality of home-grown players participating in the English professional leagues (Premier League, 2011). An integral part of the system is the testing of the physical characteristics of players (e.g., their stature and body mass, and their sprinting, agility, vertical jumping, and endurance performance) using field-based protocols, as these allow large numbers of players to be tested in a short time and the tests are reliable and valid when conducted appropriately (Hulse et al., 2013). Indeed, in England, professional academies are required to conduct physical tests on their players aged U9-U21, at least 3 times per year. The aim of testing, and the subsequent creation of a national database, is “to enable each club to measure the relative success of their own programmes and players” (Premier League, 2011, p. 65). Interestingly, debate remains regarding the utility of a physical testing programme for talent identification and development processes in football (Mendez-Villanueva and Buchheit, 2013).

A major focus of talent identification and development research in football has been on establishing whether, and if so which, physical characteristics may be associated with success, possibly due to the importance of physical attributes to excellence in match-play (Stolen et al., 2005; Faude et al., 2012). Based on a range of physical characteristics (such as stature, body mass, body composition, speed, agility, vertical jumping, power, repeated sprint ability, and endurance), researchers have differentiated more successful elite youth football players from those who were less successful (e.g., retained vs. released from an academy) at multiple age groups from U9-U21 (Visscher et al., 2006; Gil et al., 2007, 2014; Gravina et al., 2008; Lago-Penas et al., 2011, 2014; Huijgen et al., 2014; Deprez et al., 2015; Honer and Votteler, 2016; Bennett et al., 2019; Castillo et al., 2019; Patel et al., 2020). However, such studies are typically cross-sectional, and can only provide information on current, rather than future, accomplishments (Abbott and Collins, 2002; le Gall et al., 2010). As the ultimate aim is to recognize young players with the potential to become elite senior players, it is necessary to prospectively track young players into adulthood to determine their senior playing status.

Carling et al. (2012) did perform a prospective study and showed that, of 158 academy football players from France aged 13 y, those who later became senior professionals were taller, heavier, and had a higher estimated maximal oxygen uptake (from a continuous track test) than their senior non-professional counterparts. Other studies have adopted similar approaches for single (Deprez et al., 2015; Martinez-Santos et al., 2016; Honer et al., 2017; Castillo et al., 2018; Sieghartsleitner et al., 2019a,b) or several distinct age groups (le Gall et al., 2010; Gonaus and Muller, 2012; Emmonds et al., 2016). While prospective, such designs are still limited in that they do not provide insight into the changes and pattern of development of physical characteristics over the length of a player's academy career and into adulthood. Also, they fail to consider talent identification and development as an interlinked, dynamic, and non-linear process (Simonton, 1999; Williams and Reilly, 2000; Abbott et al., 2005) and perhaps implicitly suggest that talent identification and development is a short-, rather than a long-term process (Sarmento et al., 2018). Therefore, multiple observations of the same individuals across time are needed to adequately track and examine the changes and the pattern of development of dynamic variables such as physical characteristics, so that casual relationships and individual developmental trajectories can be more robustly examined (Saward et al., 2016; Fransen et al., 2017; Johnston et al., 2018). Thus, the optimal research design needs to be longitudinal and prospective. That is, longitudinally monitoring changes in elite youth players' physical characteristics over several years and subsequently determining their senior playing status would allow the changes and pattern of development of physical characteristics of the most talented players to be understood, which in turn could better support talent identification and development processes (Huijgen et al., 2009; Coutinho et al., 2016; Leyhr et al., 2019). However, to the authors' knowledge only two studies have adopted this type of design in examining the development of elite youth male football players' physical characteristics (see Roescher et al., 2010; Leyhr et al., 2018).

Leyhr et al. (2018) longitudinally examined the development of 20-m sprint and slalom agility performance in 1,134 players aged U12-U15 from a German talent center and determined their senior playing status 8 years later. Multilevel modeling revealed that development of 20-m sprint and slalom agility performance was non-linear and future senior elite players (top 5 German divisions) were quicker on a slalom agility test than future senior non-elite players throughout development, but there was no difference in the pattern of development (Leyhr et al., 2018). There were no differences in 20-m sprint times between the groups. Roescher et al. (2010) used multilevel modeling to assess the longitudinal development of interval shuttle run test performance in 130 future professional and non-professional football players aged 14–18 years from two Dutch academies. For interval shuttle run test performance there was no difference between future professionals and non-professionals between 14 and 16 y. After age 16 y, the pattern of development became different for the two groups, with future professionals outperforming future non-professionals, as the professionals continued to improve in an almost linear fashion while the non-professionals improved at a slower rate, thus the differences between groups became larger over time (Roescher et al., 2010).

These studies (Roescher et al., 2010; Leyhr et al., 2018) are valuable as they are the only studies in elite youth male football players which have adequately modeled longitudinal data and linked this with the future professional status of the players they investigated. However, both studies did have limitations. For example, player development was examined over a relatively short period of time (~4 y) when compared to the full duration of a talent development programme (~10 y). Also, neither study examined any anthropometric data, and only one or two physical performance characteristics were assessed in each study, and this may not be a broad enough representation of the physical characteristics needed to progress to professional status in football (Stolen et al., 2005). It could also be argued that the study of Roescher et al. (2010) had a relatively small sample size (*n* = 130). In addition, cross-sectional research has demonstrated differences in the physical characteristics of elite youth players based on their playing position (Deprez et al., 2015a) yet with regards to the longitudinal development of several physical characteristics in future professionals, the role of playing position remains largely unaccounted for. Finally, the studies of Leyhr et al. (2018) and Roescher et al. (2010) examined players from only one or two academies from Germany and the Netherlands, and it remains unclear whether these findings can be replicated in players from another country (such as those from the English talent development system), and replicated when player data from multiple different academies are examined.

Therefore, the purpose of the present study was to examine if elite youth male football players aged 8–19 y from the English talent development system who ultimately achieved professional status differed in stature, body mass, and physical performance (20-m sprint speed, slalom agility speed, vertical counter-movement jump with arm swing jump height, multistage fitness test distance) compared with their non-professional peers. The study also examined the longitudinal pattern of development of stature, body mass, and physical performance, and if this was different between future professionals and non-professionals, while considering the effects of playing position.

## Methods

### Participants and Sample

A total of 2,875 elite male youth football players aged 8–19 y participated in the study (Table 1). Participants were defined as elite youth football players as they were recruited from 16 professional academies in England (Swann et al., 2015). Longitudinal anthropometric and physical performance test data were collected on players between 2002 and 2013, resulting in 8,898 individual (player-occasion) data points (Mean ± SD = 3.1 ± 2.7, Range = 1–24). Playing position was also recorded at each testing session, resulting in 686, 2,563, 3,063, 1,923, and 633 individual (player-occasion) data points for goalkeepers, defenders, midfielders, forwards, and multi-positional players, respectively.

**Table 1:** Player age, stature, and body mass by future playing status (non-professional vs. professional) and age group (U9 to U19).

	**Age (decimal years)**		**Stature (cm)**		**Body mass (kg)**	
**Age group**	**Non-PROF [*n*]**	**PROF [*n*]**	**Non-PROF [*n*]**	**PROF [*n*]**	**Non-PROF [*n*]**	**PROF [*n*]**
U9	9.1 ± 0.4 [746]	9.0 ± 0.4 [144]	135.0 ± 5.5 [708]	135.0 ± 5.3 [144]	30.6 ± 3.9 [708]	30.5 ± 3.8 [144]
U10	10.1 ± 0.4 [841]	10.1 ± 0.4 [158]	140.0 ± 5.7 [802]	140.9 ± 5.6 [156]	33.9 ± 4.7 [801]	34.5 ± 4.4 [156]
U11	11.1 ± 0.3 [834]	11.1 ± 0.4 [205]	144.6 ± 6.4 [778]	146.6 ± 6.1 [195]	37.0 ± 5.6 [777]	38.5 ± 5.3 [196]
U12	12.1 ± 0.3 [892]	12.1 ± 0.4 [200]	151.6 ± 7.4 [845]	152.0 ± 7.1 [194]	41.9 ± 7.0 [845]	42.0 ± 5.6 [194]
U13	13.1 ± 0.3 [814]	13.1 ± 0.3 [231]	158.3 ± 9.1 [779]	158.5 ± 8.6 [217]	47.5 ± 8.9 [778]	47.5 ± 8.0 [217]
U14	14.1 ± 0.4 [796]	14.1 ± 0.4 [282]	167.5 ± 8.7 [755]	166.3 ± 9.4 [272]	56.5 ± 9.6 [755]	54.9 ± 9.6 [273]
U15	15.1 ± 0.4 [608]	15.1 ± 0.4 [264]	173.0 ± 7.6 [598]	173.5 ± 8.8 [262]	63.4 ± 9.1 [598]	62.8 ± 9.7 [263]
U16	16.1 ± 0.4 [402]	16.1 ± 0.4 [280]	177.0 ± 6.4 [387]	177.2 ± 6.8 [271]	68.7 ± 7.8 [394]	68.3 ± 8.2 [275]
U17	17.2 ± 0.4 [330]	17.1 ± 0.4 [280]	178.4 ± 6.5 [312]	179.8 ± 6.1 [270]	71.9 ± 7.6 [325]	72.5 ± 6.7 [276]
U18	18.0 ± 0.4 [230]	18.0 ± 0.4 [237]	178.5 ± 6.2 [224]	180.2 ± 5.8 [233]	72.5 ± 7.5 [226]	74.2 ± 7.0 [229]
U19	19.0 ± 0.3 [68]	18.9 ± 0.3 [56]	178.9 ± 5.7 [68]	179.7 ± 5.1 [55]	74.6 ± 6.4 [67]	75.7 ± 7.6 [53]

*Non-PROF, players who did not make a professional league appearance; PROF, players who made at least one professional league appearance; n, number of data points in the sample*.

Ethical approval for the study was obtained from the University Ethical Advisory Committees. Prior to taking part in the study, participants and parents/guardians were provided with a written and verbal summary outlining the purpose, procedures involved, possible risks and benefits, and the voluntary and confidential nature of the research. For participants aged 18 y or above, written informed consent was obtained. For participants under 18 y, written assent was obtained from players and written consent was obtained from their parents/guardians. Prior to every anthropometric and physical performance testing session, participants went through a health screening process to identify any reasons that may affect, or exclude them from, participation.

### Performance Testing Procedures

Participants completed a battery of anthropometric and physical performance field-tests validated in elite youth football players (Hulse et al., 2013). Firstly, the anthropometric assessments were conducted, which included the measurement of stature and body mass. Stature was measured (to the nearest 0.1 cm) using a stadiometer (Leicester Height Measure, seca ltd., England). Body mass was measured (to the nearest 0.01 kg) using portable digital scales (seca 770, seca ltd, Birmingham, UK). Subsequently, players completed four physical performance field-tests: 20-m sprint test, a 20.8 m slalom agility test, a vertical counter-movement jump with arm swing (CMJA), and the multistage fitness test (MSFT Ramsbottom et al., 1988). The testing battery took place on an indoor new-generation synthetic surface. The physical performance field-tests were preceded by a verbal explanation of the test, a standardized familiarization and a warm-up procedure. Each test was separated by a ~3-min recovery.

**Figure 1:**
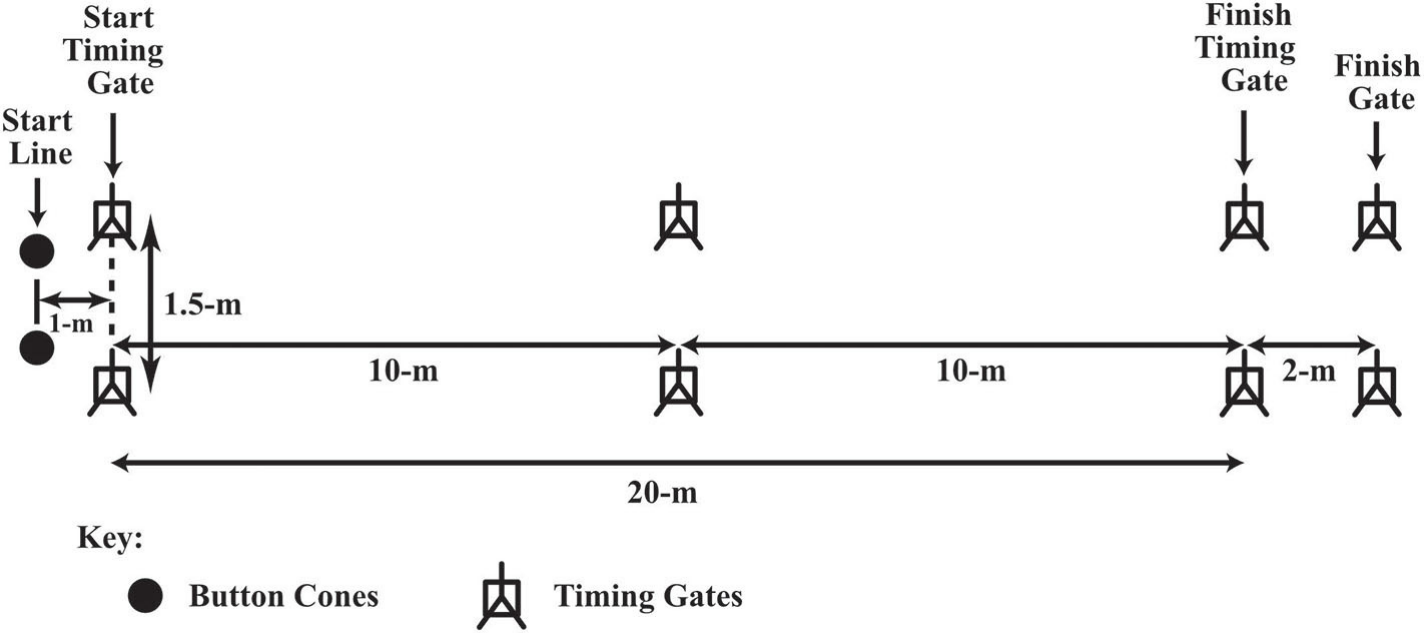
20-m sprint test layout and dimensions.

**Figure 2:**
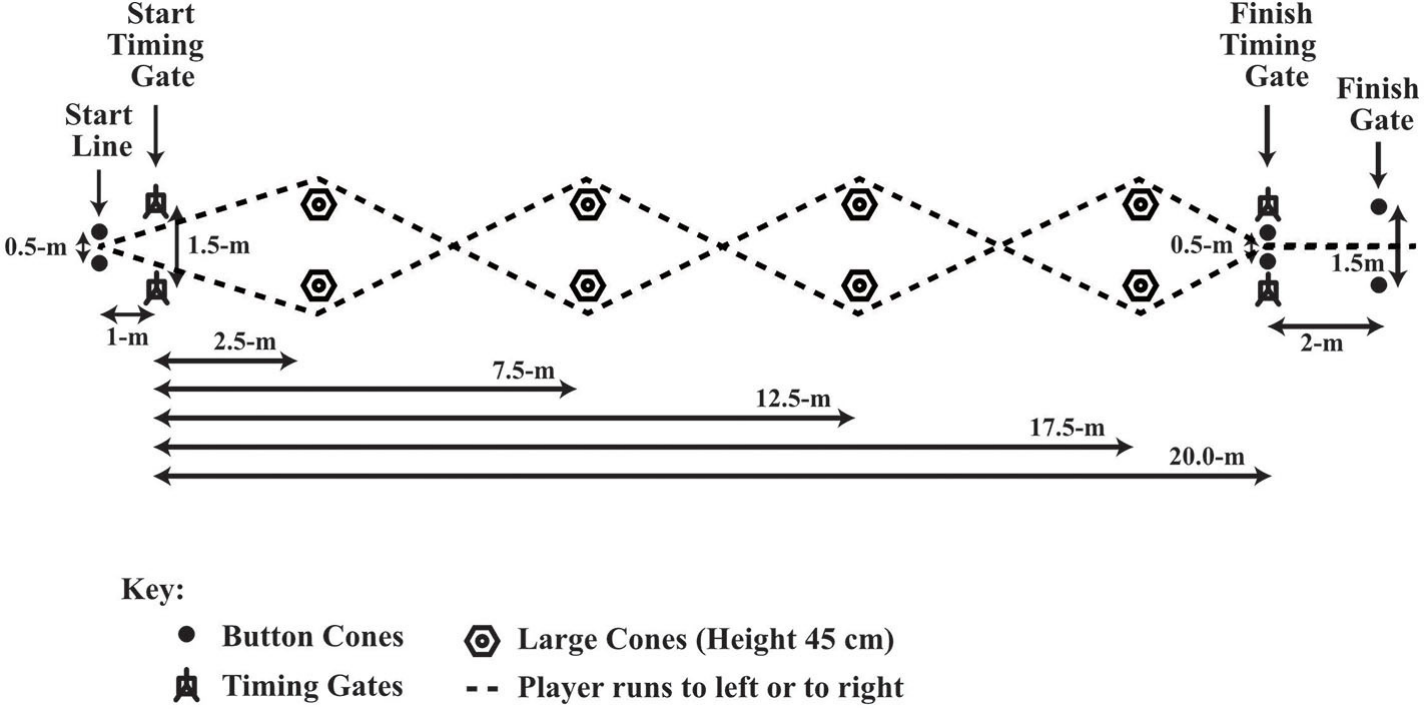
20.8-m slalom agility test layout and dimensions.

**Figure 3:**
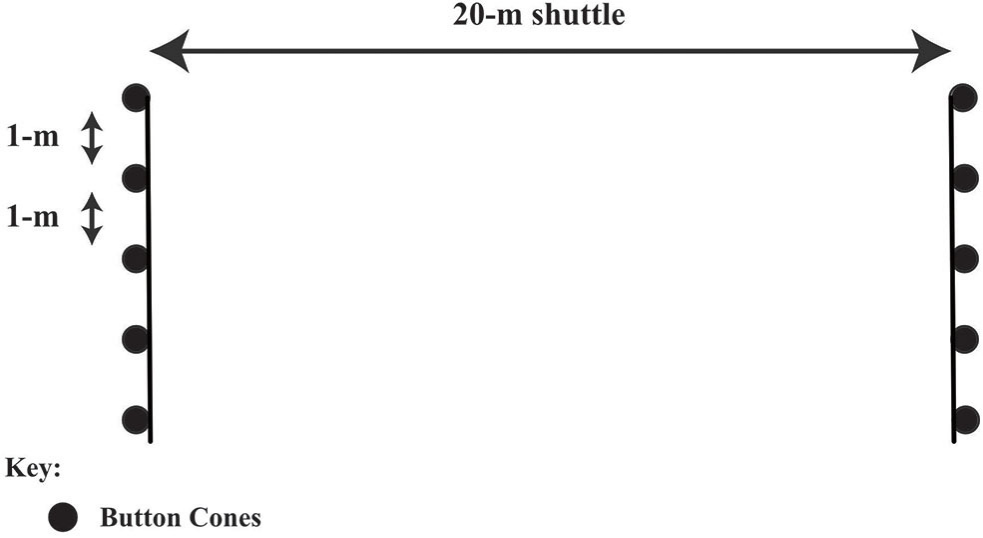
The multistage fitness test layout and dimensions.

For the 20-m sprint test (see Figure 1) participants completed two practice efforts, followed by two maximal sprints separated by a 3-min recovery period. Participants began the sprint in their own time, off their preferred foot, on a line 1-m behind the starting gate. The best time for 20-m was used in the current analysis. For the 20.8-m slalom agility test each participant completed four practice runs through the agility course (see Figure 2): two runs, where the first cone of the slalom was to their left, and two where the first cone was to their right side. Participants then performed alternate maximal efforts through each course (four in total), separated by a 3-min recovery period. Participants began their effort in their own time, off their preferred foot, on a line 1-m behind the starting gate. The agility time used in the current analysis was an aggregate (mean) of the participant's fastest left and right performance. For the 20-m sprint and slalom agility tests, times were measured to the nearest 0.01 s using infrared photoelectric cells (Newtest, Oulu, Finland, or Brower timing system, Draper, Utah, USA). Participants performed a vertical counter-movement jump with full use of their arms (Newtest Jump Mat, Oulu, Finland or SmartJump, Fusion Sport, Australia) at least 2 times, separated by a 3-min recovery period. Participants performed the multistage fitness test with an adult “pacemaker” (see Figure 3). The multistage fitness test involved running back and forth over a 20-m distance in time with an audio signal. Participants were required to reach the end of each 20-m shuttle and to place their foot on or over the line marking the 20-m length at the same time as the “bleep” audio signal sounded. The required speed started at 8.0 km.h^−1^, increased to 9.0 km.h^−1^ after 63-s and then increased by 0.5 km.h^−1^ approximately every 60-s thereafter. The test ended when a participant voluntary indicated they no longer wished to continue running or they could no longer keep pace with the audio signal on three consecutive 20-m shuttles. The final level and shuttle completed by a player was recorded and then converted to a distance which was used in the current analysis. Hulse et al. (2013) reported acceptable reliability for tests completed 7 days apart for all age groups (U9-U11, U12-U14, U15-U18), with coefficients of variation of 1.5–1.7, 2.5–2.7, and 4.4–4.6%, for the 20-m sprint test, the slalom agility test and the vertical counter-movement jump with arm swing test, respectively. The coefficient of variation for the multistage fitness test has been reported to be 2–3% (Aziz et al., 2005; Cooper et al., 2005).

### Establishing Professional Status

Subsequently, players were prospectively tracked, and their playing status determined as of 31st July 2019. A website containing information on professional football (Transfermarkt.com) was used to determine playing status, namely the number of professional league appearances (i.e., appearances in official league matches for a full-time senior professional football team) made by each player. The final sample consisted of 653 players who made at least one professional league appearance (23%) and 2,222 players who did not make an appearance. The group of professional players made 126 ± 123 (mean ± SD) (minimum = 1, maximum = 516) appearances across 65 professional leagues in Africa, America, Asia, Australasia, and Europe.

### Data Analysis

The longitudinal sample used in the present study represents a hierarchically structured data set, with measurement occasions nested within player. As such, multilevel growth curve modeling was used to examine the development of physical characteristics (MLwiN v 3.00, Bristol, U.K.). Unlike traditional longitudinal data analysis techniques, such as the repeated measures ANOVA, multilevel modeling does not require the same number of measurement occasions per individual. Moreover, the temporal spacing of measurements may vary between players (Rasbash et al., 2017). Hence this statistical technique was suited to the current data structure. A multilevel model describes the underlying trends of a particular component in the population (fixed part), and also models the unexplained variation around the mean trend for that component (random part) (Twisk, 2003).

Following the guidelines of Rasbash et al. (2017) a two-level hierarchical structure was defined, with measurement occasion (level 1) nested within player (level 2), with a given physical characteristic as the continuous response variable. Subsequently, relevant parameters were systematically considered to observe their effect on explaining and partitioning variation in player development. Parameters were accepted or rejected based on changes in model fit, as indicated by differences in −2 loglikelihood, and the effect of explanatory variables on the response variable, as indicated by z-scores. From an empty model (i.e., the response variable and a fixed intercept), the intercept was allowed to randomly vary and a linear age term (centered at 13.0 y) was added. This forms the simplest multilevel growth model. From this simple multilevel growth model, quadratic, and cubic age terms were considered to allow for non-linearity of development. Variance between players' development rates was then considered by allowing the age terms to randomly vary. Subsequently a series of fixed explanatory variables were considered in turn, in the following order: professional playing status, playing position, and interactions between age, playing status, and playing position. Following each analysis, the assumption that the player-level random effects followed a bivariate normal distribution with zero means, was checked (Rasbash et al., 2017). Statistical significance was accepted at the 95% confidence level (*p* < 0.05). The size of the effects when comparing future playing status (professionals vs. non-professionals) and playing position (goalkeeper, defender, midfielder, forward, multi-positional) were examined using Cohen's *d* adapted for multilevel modeling (Feingold, 2019). Effect sizes were evaluated using <0.2, 0.2, 0.5, and 0.8 as the boundaries for trivial, small, moderate and large effects, respectively (Cohen, 1992). Mean ± SD were used to describe the average and variability of data, unless stated otherwise.

In terms of interpreting the outputs from multilevel models, the *Fixed Effects* part of the models show the expected characteristics (underlying trends) of professional and non-professional players at age 13.0 y for the different playing positions (see **Table 3**). At age 13.0 y, the average non-professional forward player is predicted to be 158.75 cm tall, weigh 48.80 kg, sprint 20-m straight at 6.098 m.s^−1^, complete the slalom agility test at 4.647 m.s^−1^, jump 36.645 cm on the vertical counter-movement jump with arm swing test, and complete 1,759 m during the multistage fitness test (see 4th row of data in **Table 3**). It is also possible to estimate the development of characteristics with age for players of future professional and non-professional playing standards and different playing positions, using the fixed coefficients from **Table 3**. For example, the prediction equation for vertical counter-movement jump with arm swing test performance for a 16.5-year-old professional forward is: Forward [β0 for forward) + (β1^*^ Age centered at 13.0 y) + (β2 ^*^ Age centered at 13.0 y^2^) + (β3 ^*^ Age centered at 13.0 y^3^) + (β4 ^*^ Professional) + (β5^*^ Age centered at 13.0 y^*^Professional), which is 36.645 cm + (2.624 cm ^*^ 3.5 y) + (0.012 cm ^*^ 12.3 y) + (−0.030 cm ^*^ 42.9 y) + (0.777 cm ^*^ 1)+ (0.191 cm ^*^ (3.5 y^*^1)] = 46.135 cm. The *Random Effects* section of **Table 3** allows the variation in development between players to be described (i.e., inter-individual differences). For example, for the development of multistage fitness test performance, the average distance for a 13.0 y for a professional midfielder is 1,876 m (1,829 m + 47 m, see **Table 3**, *Fixed Effects*), but with an intercept SD of 197 m (see **Table 3**, *Random Effects*) the coverage range within which 95% of professional midfielders intercepts are expected to lie at age 13.0 y can be estimated as 1,490–2,262 m [1,876 ± (1.96^*^197 m)]. Furthermore, multistage fitness test performance is predicted to increase by 132 m per year for the average player (see **Table 3**, *Fixed Effects*, Age^1^), but with a slope (Age^1^) SD of 40 m (see **Table 3**, *Random Effects*), the coverage range within which 95% of players' growth rates are expected to lie can be estimated as 54–210 m per year [132 ± (1.96^*^40 m)].

## Results

### Descriptive Data

Tables 1, 2 describe the anthropometric and physical performance characteristics of the 2,875 elite youth football players examined in the study according to age group and future professional or non-professional playing status.

**Table 2:** Player physical performance test results by future playing status (non-professional vs. professional) and age group (U9 to U19).

	**Speed over 20 m (m.s**^****−1****^**)**		**Speed over the slalom agility test (m.s**^****−1****^**)**		**Vertical counter-movement jump with arm swing (cm)**		**Multistage fitness test distance completed (m)**	
**Age group**	**Non-PROF [*n*]**	**PROF [*n*]**	**Non-PROF [*n*]**	**PROF [*n*]**	**Non-PROF [*n*]**	**PROF [*n*]**	**Non-PROF [*n*]**	**PROF [*n*]**
U9	5.39 ± 0.24 [711]	5.46 ± 0.21 [144]	4.13 ± 0.25 [706]	4.21 ± 0.26 [144]	27.5 ± 4.0 [720]	28.0 ± 4.1 [144]	1,288 ± 275 [194]	880 ± 57 [2]
U10	5.52 ± 0.25 [801]	5.61 ± 0.25 [156]	4.28 ± 0.25 [801]	4.31 ± 0.24 [155]	29.1 ± 4.4 [811]	30.1 ± 4.4 [156]	1,431 ± 282 [221]	1,238 ± 286 [8]
U11	5.66 ± 0.26 [769]	5.72 ± 0.23 [196]	4.41 ± 0.27 [778]	4.45 ± 0.26 [195]	31.6 ± 4.5 [788]	31.7 ± 4.0 [196]	1,542 ± 276 [167]	1,287 ± 291 [6]
U12	5.80 ± 0.28 [841]	5.90 ± 0.28 [193]	4.50 ± 0.27 [837]	4.53 ± 0.30 [194]	32.8 ± 4.7 [849]	33.6 ± 4.4 [194]	1,562 ± 258 [189]	1,614 ± 330 [30]
U13	6.02 ± 0.31 [774]	6.06 ± 0.30 [213]	4.60 ± 0.28 [773]	4.63 ± 0.27 [212]	36.0 ± 5.3 [784]	36.5 ± 5.0 [219]	1,705 ± 289 [170]	1,729 ± 328 [28]
U14	6.29 ± 0.35 [744]	6.29 ± 0.33 [268]	4.75 ± 0.32 [741]	4.80 ± 0.30 [262]	39.4 ± 6.0 [737]	39.6 ± 5.0 [270]	1,912 ± 258 [164]	2,011 ± 281 [51]
U15	6.50 ± 0.34 [571]	6.55 ± 0.30 [256]	4.87 ± 0.27 [573]	4.98 ± 0.27 [254]	42.4 ± 5.4 [565]	43.0 ± 5.8 [251]	2,085 ± 250 [87]	2,149 ± 263 [41]
U16	6.64 ± 0.29 [382]	6.70 ± 0.28 [263]	4.94 ± 0.27 [386]	5.10 ± 0.29 [269]	44.2 ± 5.3 [385]	46.4 ± 5.8 [271]	2,163 ± 287 [63]	2,281 ± 246 [48]
U17	6.69 ± 0.30 [308]	6.75 ± 0.27 [266]	5.03 ± 0.28 [311]	5.14 ± 0.28 [264]	44.9 ± 4.7 [308]	47.0 ± 6.2 [264]	2,232 ± 282 [43]	2,265 ± 202 [48]
U18	6.73 ± 0.24 [213]	6.78 ± 0.25 [220]	5.01 ± 0.30 [216]	5.12 ± 0.28 [218]	45.5 ± 5.2 [213]	46.8 ± 5.5 [214]	2,364 ± 239 [18]	2,234 ± 210 [24]
U19	6.67 ± 0.30 [63]	6.75 ± 0.23 [50]	4.89 ± 0.28 [64]	5.05 ± 0.26 [50]	46.3 ± 7.1 [64]	46.9 ± 6.1 [50]	-	2,030 ± 184 [2]

*Non-PROF, players who did not make a professional league appearance; PROF, players who made at least one professional league appearance; n, number of data points in the sample*.

### Stature, Body Mass, and Future Professional Playing Status

**Figure 4:**
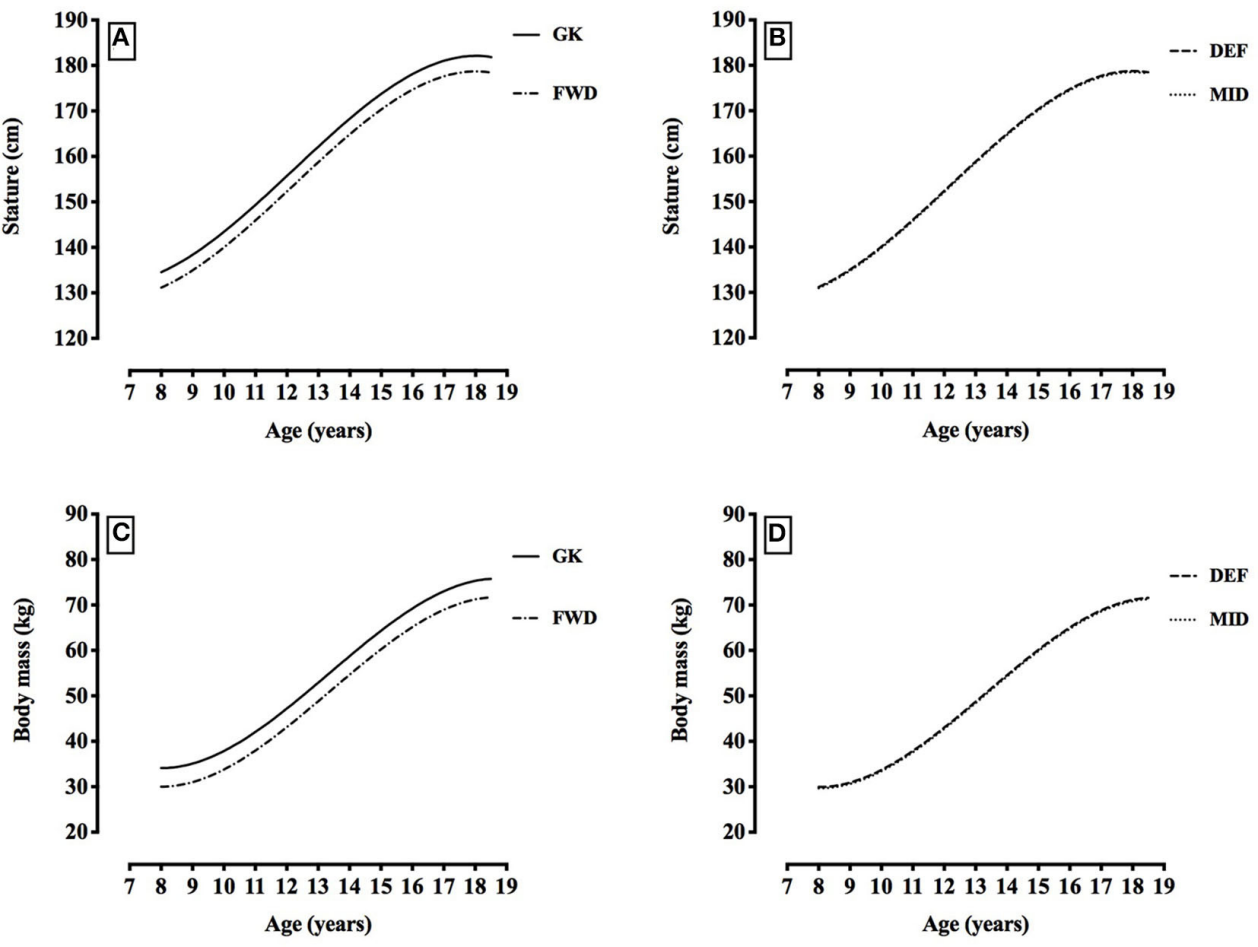
Changes in stature and in body mass with age by playing position [stature: **(A)** goalkeepers (GK) and forwards (FWD), **(B)** defenders (DEF) and midfielders (MID); body mass: **(C)** goalkeepers (GK) and forwards (FWD); **(D)** defenders (DEF) and midfielders (MID)]. Data is based on the fixed parameter estimates from the models described in Table 3.

Table 3 shows the final multilevel models describing the longitudinal development of anthropometric characteristics (stature and body mass). No differences in stature and body mass were evident when the non-professional and professional players were compared (indicated by no parameter estimate in Table 3 for the “Professional” variable, as, when added to the model, the ratio between the “Professional” variable parameter estimate and its associated standard error (z-score) did not achieve significance (*p* > 0.05), and the fit of the model was not improved by the inclusion of the variable, hence its omission). Also, no differences were evident in the longitudinal pattern of development of stature and body mass across age, when the non-professional and professional players were compared (indicated by no parameter estimates in Table 3 for the interaction “Age^1*^Professional” and “Age^2*^Professional” variables). However, the analysis in Table 3 does suggest that the longitudinal development of stature and body mass with age was not linear (as indicated by the significant “Age^2^” and “Age^3^” terms which add curvature to the model fit—see Figure 4 for an illustration of this cubic pattern of development). There were inter-individual differences in stature and body mass at each age (random intercept), and inter-individual differences in the rate of change of stature and body mass with age (random slope for Age^1^). The model suggests that 95% of 13.0-year-old players would have a stature within ±14.2 cm of the statures reported in the first five rows of Table 3 (see the data analysis section for how this coverage range is calculated). For body mass, the equivalent boundary was calculated to be ±13.7 kg. In terms of the variation in growth rates, the predicted slope or annual change in stature for 95% of players from 13.0 to 14.0 y would be between 3.3 and 9.0 cm per year. For body mass, the equivalent boundaries would be between 3.2 and 8.5 kg per year.

**Table 3:** Multilevel models for the development of physical characteristics in elite youth football players aged 8–19 y.

**Parameter**	**Stature (cm)**	**Body mass (kg)**	**20-m speed (m^**.**^s^**−1**^)**	**Slalom agility speed (m^**.**^s^**−1**^)**	**CMJA (cm)**	**MSFT (m)**
	**Estimate (SE)**	**Estimate (SE)**	**Estimate (SE)**	**Estimate (SE)**	**Estimate (SE)**	**Estimate (SE)**
**FIXED EFFECTS**						
Goalkeeper	162.17 (0.41)^a^^,^^b^^,^^c^^,^^d^	52.88 (0.43)^a^^,^^b^^,^^c^^,^^d^	5.860 (0.017)^a^^,^^b^^,^^c^^,^^d^	4.474 (0.017)^a^^,^^b^^,^^c^^,^^d^	34.948 (0.306)^a^^,^^b^^,^^c^^,^^d^	1,524 (28)^a^^,^^b^^,^^c^^,^^d^
Defender	158.80 (0.17)^b^^,^^e^	48.72 (0.18)^e^	6.039 (0.009)^c^^,^^e^	4.604 (0.009)^c^^,^^e^	35.873 (0.154)^c^^,^^e^	1,802 (15)^c^^,^^e^
Midfielder	158.54 (0.16)^a^^,^^e^	48.44 (0.17)^c^^,^^e^	6.034 (0.008)^c^^,^^e^	4.603 (0.008)^c^^,^^e^	35.668 (0.144)^c^^,^^e^	1,829 (14)^c^^,^^e^
Forward	158.75 (0.18)^e^	48.80 (0.19)^b^^,^^e^	6.098 (0.010)^a^^,^^b^^,^^d^^,^^e^	4.647 (0.010)^a^^,^^b^^,^^d^^,^^e^	36.645 (0.173)^a^^,^^b^^,^^d^^,^^e^	1,759 (17)^a^^,^^b^^,^^e^
Multipositional	158.61 (0.19)^e^	48.77 (0.21)^e^	6.030 (0.012)^c^^,^^e^	4.599 (0.012)^c^^,^^e^	35.855 (0.208)^c^^,^^e^	1,779 (27)^e^
Age^1^	6.356 (0.048)	5.845 (0.052)	0.193 (0.003)	0.111 (0.002)	2.624 (0.050)	132 (3)
Age^2^	−0.153 (0.008)	0.073 (0.009)	−0.001 (0.001)	−0.004 (0.001)	0.012 (0.009)	–
Age^3^	−0.064 (0.002)	−0.069 (0.002)	−0.002 (0.001)	–	−0.030 (0.002)	–
Professional	–	–	0.057 (0.012)	0.034 (0.013)	0.777 (0.213)	47 (19)
Age^1^ * Professional	–	–	–	0.011 (0.004)	0.191 (0.067)	–
Age^2^ * Professional	–	–	–	0.003 (0.001)	–	–
**RANDOM EFFECTS**						
Intercept SD	7.27	6.99	0.21	0.20	4.09	197
Slope (Age^1^) SD	1.45	1.36	0.03	0.03	0.66	40
Residual SD	1.04	1.54	0.16	0.17	2.58	165
Δ−2 loglikelihood (df)	31,453 (10)	27,655 (10)	15,055 (11)	10,058 (12)	13,413 (12)	3,789 (9)

*Parameters accepted at p < 0.05. Independent (intercept) estimates (at 13.0 y) for each playing position displayed. Between-position differences*:

a *significant difference vs. Defender*,

b *vs. Midfielder*,

c *vs. Forward*,

d *vs. Multipositional*,

e vs. Goalkeeper. Centered at 13.0 y, Age^1^, Age^2^, and Age^3^ refer to the linear, quadratic, and cubic age terms, respectively. Professional: the effect of being professional at age 13.0 y. Age^1^ * Professional: interaction between linear age and professional. Age^2^ * Professional: interaction between quadratic age and professional. Intercept SD: inter-individual variation at age 13.0 y. Slope (Age^1^) SD: inter-individual variation in linear growth rates. Residual SD: within-individual variation. Δ−2 loglikelihood (df) is the change in model fit, and associated degrees of freedom, from the empty model to the final model. CMJA, vertical counter-movement jump with arm swing; MSFT, multistage fitness test.

### Physical Performance Characteristics and Future Professional Playing Status

**Figure 5:**
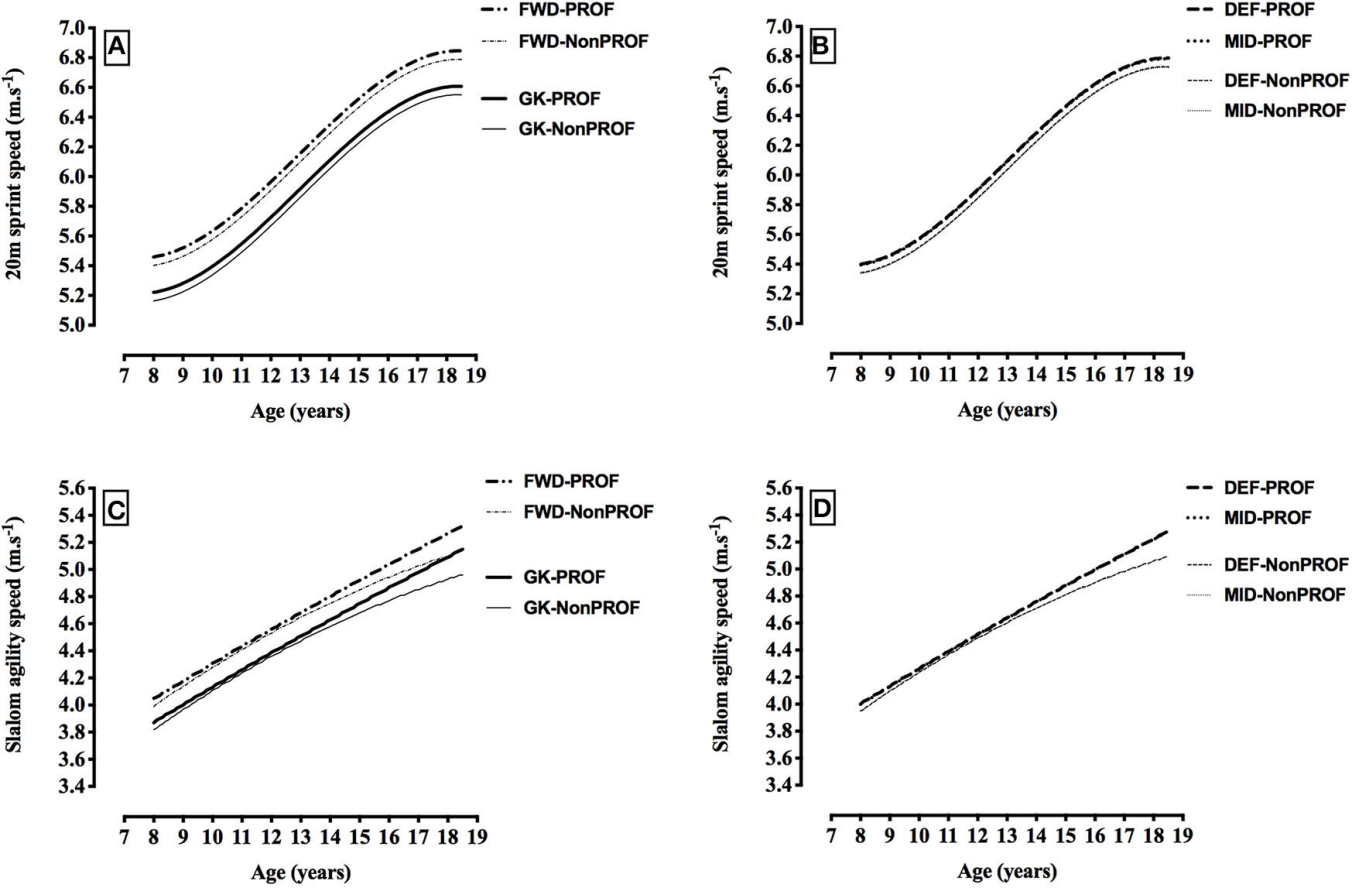
Changes in 20-m sprint speed (m.s^−1^) and slalom agility speed (m.s^−1^) with age, by playing position and future playing standard [20-m sprint speed: **(A)** goalkeepers (GK) and forwards (FWD), **(B)** defenders (DEF) and midfielders (MID; slalom agility speed: **(C)** goalkeepers (GK) and forwards (FWD); **(D)** defenders (DEF) and midfielders (MID); PROF, players who made at least one professional league appearance; Non-PROF, players who DID NOT make any professional league appearances)]. Data is based on the fixed parameter estimates from the models described in Table 3. (Please see alternative representations of this data in the Supplemental Material).

Table 3 also shows the final multilevel models describing the longitudinal development of physical performance characteristics (20-m sprint test, slalom agility test, vertical counter-movement jump with arm swing test, and multistage fitness test) in the sample of academy football players. Future professional players were faster in a 20-m sprint than future non-professional players throughout their development by 0.057 m.s^−1^ (*p* < 0.001, see “Professional” parameter estimate, Table 3; *d* = 0.2). This would equate to a 0.02–0.04 s faster 20-m sprint time in the professional players compared to the non-professional players (with the variation relating to age). Age-related changes in 20-m sprint test performance were non-linear (indicated by the significant “Age^2^” and “Age^3^” terms in Table 3). The pattern of development was the same in the non-professional and professional players, and the non-linear but parallel pattern of development between the two player categories is evident in Figures 5A,B. There were inter-individual differences in 20-m sprint test performance at each age (random intercept), and inter-individual differences in the rate of change of 20-m sprint test performance with age (random slope for Age^1^). The model suggests that 95% of 13.0-year-old non-professional players would have a 20-m sprint test performance within ±0.41 m.s^−1^ of the speeds reported in the first five rows of Table 3 (add 0.057 m.s^−1^ to the values in the first five rows for the “Professional” group), which equates to a variation in 20-m sprint time of ±0.21–0.26 s. In terms of the variation in the rate of change of 20-m sprint test performance with age, the predicted slope or annual change in speed for 95% of 13.0- to 14.0-year-old players would be between 0.13 and 0.25 m.s^−1^ per year (which equates to a variation in the annual rate of change in 20-m sprint time of ±0.08–0.14 s per year).

The longitudinal pattern of development in slalom agility test performance was non-linear, and differed between the non-professional and professional players (indicated by the significant “Age^1^,” “Age^2^,” “Professional,” “Age^1*^Professional,” and “Age^2*^Professional” terms in Table 3, and illustrated in Figures 5C,D). At age 9.0 professional players were not faster on the slalom agility test (*p* = 0.143, *d* < 0.2), yet by age 12.0 professional players were significantly (*p* = 0.048), although trivially (*d* < 0.2), faster by 0.026 m.s^−1^ (equivalent to 0.03 s) than non-professional players, and by age 18.0 this difference had grown to 0.164 m.s^−1^ (equivalent to 0.14 s) (*p* < 0.001, *d* = 0.5). Professional players' annual rate of improvement was maintained at 0.11–0.13 m.s^−1^ per year throughout their development, but in the non-professional players the rate of improvement gradually declined as they got older (from 0.15 m.s^−1^ per year at 8.0 to 9.0 years of age to 0.08 m.s^−1^ per year at 17.0 to 18.0 years of age). There were inter-individual differences in slalom agility test performance at each age (random intercept), and inter-individual differences in the rate of change of slalom agility test performance with age (random slope for Age^1^). The model suggests that 95% of 13.0-year-old non-professional players would have a slalom agility test performance within ±0.39 m.s^−1^ of the speeds reported in the first five rows of Table 3 (add 0.034 m.s^−1^ to the values in the first five rows for the “Professional” group), which equates to a variation in slalom agility time of ±0.37**–**0.44 s. In terms of the variation in the rate of change of slalom agility test performance with age, the predicted slope, or annual change in speed for 95% of 13.0- to 14.0-year-old players would be between 0.05 and 0.18 m.s^−1^ per year (which equates to a variation in the annual rate of change in slalom agility time of ±0.05–0.18 s per year).

**Figure 6:**
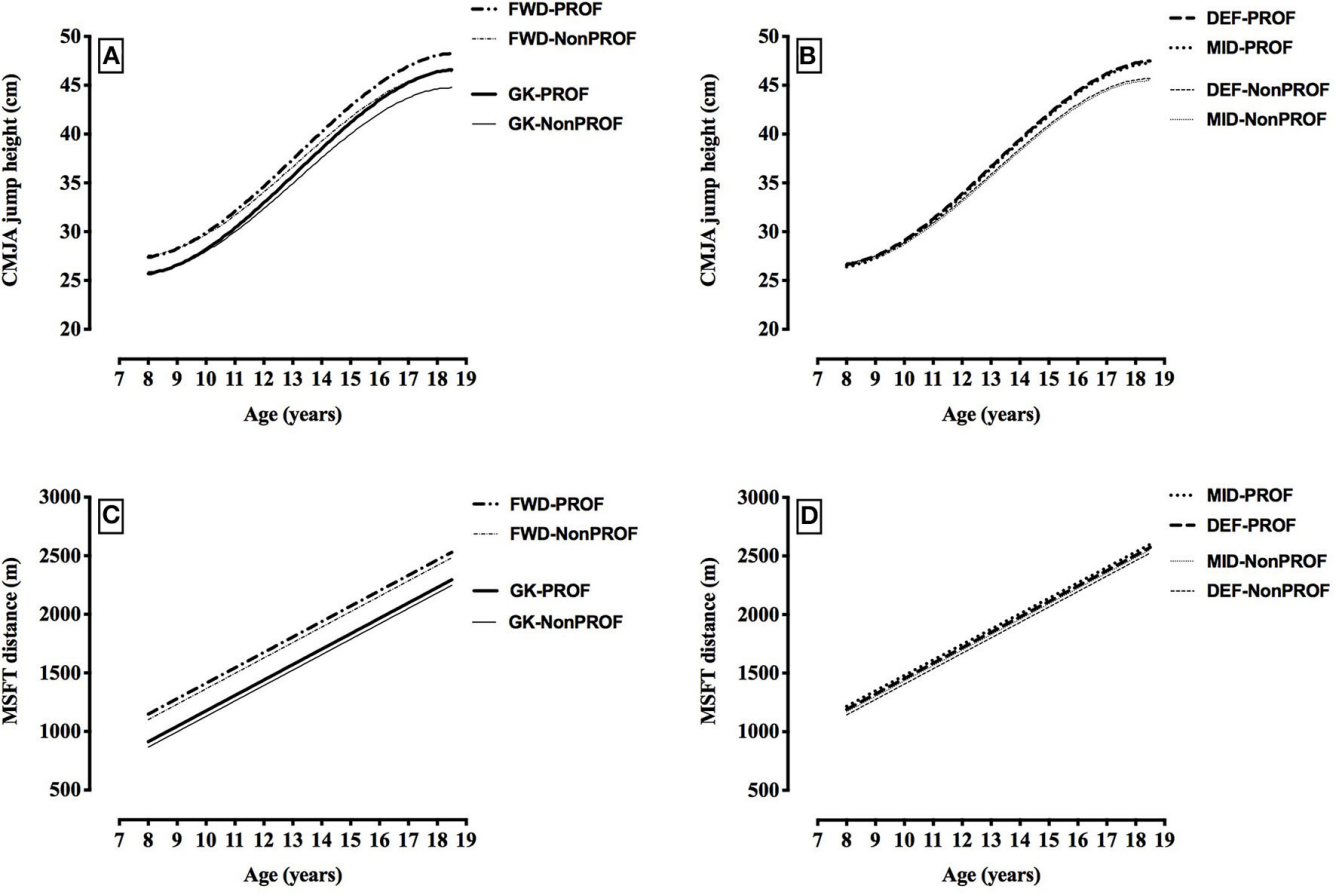
Changes in vertical counter-movement jump with arm swing (CMJA) height (cm) and multistage fitness test (MSFT) distance (m) with age, by playing position and playing standard (vertical counter-movement jump with arm swing: **(A)** goalkeepers (GK) and forwards (FWD), **(B)** defenders (DEF) and midfielders(MID); multistage shuttle run test distance: **(C)** goalkeepers (GK) and forwards (FWD); **(D)** defenders (DEF) and midfielders(MID); PROF, players who made at least one professional league appearance; Non-PROF, players who DID NOT make any professional league appearances). Data is based on the fixed parameter estimates from the models described in Table 3. (Please see alternative representations of this data in the Supplemental Material).

The longitudinal pattern of development in vertical counter-movement jump with arm swing test performance was non-linear, and differed between the non-professional and professional players (indicated by the significant “Age^1^,” “Age^2^,” “Age^3^,” “Professional,” and “Age^1*^Professional” terms in Table 3, and illustrated in Figures 6A,B). At age 9.0 there was no difference in vertical counter-movement jump with arm swing test performance between the non-professional and professional players (*p* = 0.968, *d* < 0.2), yet by age 12.0 the professional players jumped significantly (*p* = 0.007), although trivially (*d* < 0.2) higher by 0.6 cm, and by age 18.0 this difference had grown to 1.7 cm (*p* < 0.001, *d* = 0.3). Professional players' annual rate of improvement was always higher than the non-professional players, by 0.2 cm per year. There were inter-individual differences in vertical counter-movement jump with arm swing test performance at each age (random intercept), and inter-individual differences in the rate of change in vertical counter-movement jump with arm swing test performance with age (random slope for Age^1^). The model suggests that 95% of 13.0-year-old non-professional players would have a vertical counter-movement jump with arm swing jump height within ±8.0 cm of the heights reported in the first five rows of Table 3 (add 0.777 cm to the values in the first five rows for the “Professional” group). In terms of the variation in the rate of change of vertical counter-movement jump with arm swing test jump height with age, the predicted slope or annual change in jump height for 95% of 13.0–14.0-year-old players would be between 13.0- to 14.0-year-old cm per year.

Future professional players ran 47 m further on the multistage fitness test than future non-professional players throughout their development (*p* = 0.014, see “Professional” parameter estimate, Table 3; *d* = 0.2). The longitudinal pattern of development in multistage fitness test performance with age was the same in the non-professional and professional players, and the linear (indicated by the significant “Age^1^” term in Table 3) but parallel pattern of development between the two player categories is evident in Figures 6C,D. There were inter-individual differences in multistage fitness test performance at each age (random intercept), and inter-individual differences in the rate of change of multistage fitness test performance with age (random slope for Age^1^). The model suggests that 95% of 13.0-year-old players would complete a multistage fitness test distance within ±386 m of the distances reported in the first five rows of Table 3 (add 47 m to the values in the first five rows for the “Professional” group). In terms of the variation in the rate of change of multistage fitness test performance with age, the predicted slope or annual change in distance for 95% of 13.0- to 14.0-year-old players would be between 54 and 210 m per year.

### Positional Differences

Throughout development, goalkeepers were 3.4–3.6 cm taller (all *p* < 0.001, *d* = 0.3–0.5) and 4.1–4.4 kg heavier (all *p* < 0.001, *d* = 0.4–0.6) than all other positions, defenders were 0.3 cm taller than midfielders (*p* = 0.028, *d* < 0.2), and forwards were 0.4 kg heavier than midfielders (*p* = 0.025, *d* < 0.2) (see Table 3, Figure 4). Figure 4 displays the fixed effects from Table 3 to illustrate the predicted age-related changes in stature and body mass for different playing positions. For 20-m sprint speed, goalkeepers were 0.17–0.24 m.s^−1^ slower than all other positions (all *p* < 0.001, *d* = 0.5–0.9), and forwards were 0.06–0.24 m.s^−1^ faster than all other positions (all *p* < 0.001, *d* = 0.2–0.9) (see Table 3, Figure 5). For slalom agility speed, goalkeepers were 0.13–0.17 m.s^−1^ slower than all other positions (all *p* < 0.001, *d* = 0.4–0.7), and forwards were 0.04–0.17 m.s^−1^ faster than all other positions (all *p* < 0.001, *d* = 0.1–0.7), (see Table 3, Figure 5). For vertical counter-movement jump with arm swing test performance, goalkeepers did not jump as high as all other positions by 0.72–1.70 cm (*p* < 0.001—*p* = 0.026, *d* = 0.1–0.4), and forwards jumped 0.77–1.70 cm higher than all other positions (all *p* < 0.001, *d* = 0.1–0.4) (see Table 3, Figure 6). For multistage fitness test distance, goalkeepers did not run as far as all other positions by 235–305 m (all *p* < 0.001, *d* = 0.7–1.2), and forwards did not run as far as defenders by 23 m (*p* = 0.048, *d* < 0.2) and midfielders by 50 m (*p* = 0.001, *d* = 0.2) (see Table 3, Figure 6).

## Discussion

This study examined if elite youth male football players aged 8–19 y from the English talent development system who ultimately achieved professional status differed in stature, body mass, and physical performance (20-m sprint speed, slalom agility speed, vertical counter-movement jump with arm swing jump height, multistage fitness test distance) compared with their non-professional peers. The study also examined the longitudinal pattern of development of stature, body mass, and physical performance, and if this was different between future professionals and non-professionals, while considering the effects of playing position. The key findings were that from 12.0 the future professionals performed better in slalom agility and vertical counter-movement jump with arm swing tests than future non-professionals, and improved at a faster rate, so that by age 18.0 the differences in slalom agility and vertical counter-movement jump with arm swing test performance were 0.14 s and 1.7 cm, respectively. In addition, future professional players were faster (by 0.02–0.04 s on the 20-m sprint) and ran further in the multistage fitness test (by 47 m) than future non-professional players throughout their development, but there were no differences in stature or body mass during development between future professionals and future non-professionals. Furthermore, whereas multistage fitness test performance improved linearly with age, the development of all other physical characteristics was non-linear. The study also quantified the inter-individual differences in physical characteristics between all players, and also found that there were differences in physical characteristics when playing positions were compared.

The finding of physical performance differences between future professional and non-professional players during their development over the full duration of a talent identification and development programme (~10 y) confirms and extends the findings of the other two longitudinal-prospective studies in the field. Leyhr et al. (2018) noted that among the 1,134 players they investigated, the 12.8% who became elite senior players were quicker by 0.07 s than their non-elite peers in a 19.5 m slalom agility test, in comparison with the 0.03–0.14 s faster 20.8 m slalom agility time (the variation being related to age) in the present study. For endurance performance Roescher et al. (2010) found that, although there was no difference between future professional and future non-professional players in the distance run in an interval shuttle run test between the ages of 14–16 y, at age 17–18 y professionals ran ~220 m further than non-professionals. Similarly, the present study showed that future professionals ran 47 m more in the multistage fitness test than non-professional players. Therefore, consistent with previous research studies, the present study confirms that future professional football players perform better on slalom agility and endurance tests, compared with future non-professional players.

The present study also elaborates on these earlier findings by showing that future professionals sprinted faster and jumped higher than future non-professionals during their development. Future professionals were found to be 0.02–0.04 s quicker than non-professionals in the 20-m sprint test, and this is the first longitudinal-prospective study to show that future professionals jump higher than future non-professionals (up to 1.8 cm on the counter-movement jump with arm swing jump test, depending on age). It should be acknowledged that Leyhr et al. (2018) did not find any differences between future elite and non-elite players in 20-m sprint test performance, but they only considered U12-U15 age groups, and that cross-sectional-prospective studies have distinguished between playing standards based on jumping performance (for example, le Gall et al., 2010). Therefore, there seems good evidence from the current study to suggest that physical performance is better in future professional players during their development compared with future non-professionals, when examined over the full duration of a talent identification and development programme (~10 y).

While physical performance may be generally better in the future professional players as they grow and age, it is potentially really important from a talent identification and development perspective to describe and understand the longitudinal pattern of development of physical performance characteristics such as 20-m sprint speed, slalom agility speed, counter-movement jump with arm swing jump height and multistage fitness test distance. The present study found a difference in the longitudinal pattern of development of slalom agility and counter-movement jump with arm swing test performance when the future professional and non-professional players were compared. The professional players' annual rate of improvement on the counter-movement jump with arm swing test was always higher than the non-professional players by 0.2 cm per year and while professionals maintained their annual rate of improvement on the slalom agility test at 0.11–0.13 m.s^−1^ per year, the future non-professionals' rate of improvement gradually declined as they got older compared to future professionals. Thus, from 12.0 y and older the differences between future professional and non-professional players widened (see Figures 5C,D, 6A,B), so that by age 18.0 the difference on the slalom agility test was 0.14 s and the difference on the CMJA test was 1.7 cm, representing meaningful effects of small and moderate, respectively. In contrast, although Leyhr et al. (2018) also found that future elite players performed better on a slalom agility test than future non-elite players and that the pattern of development was non-linear, the pattern of development between their groups was not different (i.e., was parallel). Possible reasons for the apparent discrepancies in findings between this study and the present one may be due to the difference in the length of development period examined, variations in testing protocols or cultural differences in training across countries, and in different academies. In only one other longitudinal-prospective study has a difference in pattern of development been observed between future professional and non-professional players and this was for an intermittent endurance test, and this study was potentially limited by a small sample size (Roescher et al., 2010). Improvement on the 20-m sprint test in the present study was non-linear, and future professionals were consistently 0.057 m.s^−1^ faster than non-professionals, but the groups displayed parallel patterns of development as they aged. Leyhr et al. (2018) also found that development of 20-m sprint test performance was non-linear, however, unlike the current study they found no differences between future elite and non-elite players during development. The present study found that improvements in multistage fitness test performance were linear, and that future professionals consistently ran 47 m further than non-professional players, with the groups displaying parallel patterns of development throughout age. Therefore, based on findings from the current study, the longitudinal pattern of development of slalom agility and counter-movement jump with arm swing test performance is different in future professional players compared with future non-professionals, when examined over the full duration of a talent identification and development programme (~10 y). For 20-m sprint speed and multistage fitness test distance the longitudinal patterns of development for the future professional and non-professionals groups were parallel throughout age. Also, while multistage fitness test performance improved linearly with age, the development of all other physical characteristics was non-linear.

It is unclear why for the counter-movement jump with arm swing and slalom agility tests the future professional players showed accelerated improvements in performance compared to non-professionals, yet for multistage fitness and 20-m sprint tests although future professionals were better than non-professionals throughout development the difference between the groups remained consistent (and parallel) and an accelerated development in sprint and endurance performance in the future professionals was not observed. Factors such as later physical maturation of future professional players, fewer injuries in these players or a better attitude to training, and performance improvement could play a role. Another possible explanation is that the slalom agility and vertical counter-movement jump with arm swing tests require better neuro-muscular or coordinative abilities, compared to slightly “simpler” tests such as the 20-m sprint and the multistage shuttle test (Sheppard and Young, 2006; Deprez et al., 2015b), and/or that the slalom agility and jumping tasks are more reflective of the complex movement patterns required during match-play. The implications for talent identification and development are that while all future professional players need to be fast, agile, good vertical jumpers, and endurance fit from an early age, perhaps slalom agility, and vertical jumping offer enhanced opportunities for accelerated development and so are particularly important because as they age future professionals may increasingly be required, and be able, to produce complex movement patterns that closely reflect motor patterns in football. Such accelerated improvements may allow them to gain advantages during training and match-play as they age, which in turn may help enhance the likelihood of their retention and progression and ultimate development into successful adult players.

Previous studies have longitudinally examined the development of anthropometric characteristics such as stature and body mass in elite youth football players (Mirkov et al., 2010; Fransen et al., 2017), and others have prospectively tracked elite youth football players into adulthood to examine the relationship between anthropometric characteristics and senior playing standard (le Gall et al., 2010; Carling et al., 2012; Deprez et al., 2015; Emmonds et al., 2016). However, the present investigation is the first longitudinal and prospective study to simultaneously examine the development of stature and body mass and its relationship with senior playing standard. No differences were found in the present study in the stature and body mass of the future professional and non-professional players, or in the longitudinal pattern of development of these characteristics between the groups, suggesting these are not key distinguishing factors in the development of future professional players within English academies. This is consistent with previous work which followed 443 English academy football players aged U12-U18 into adulthood and showed no differences in stature and body mass between players who turned, vs. those who did not turn, professional, from any of the age groups examined (Emmonds et al., 2016). Interestingly, in a study more relevant to junior rather than senior accomplishments, with a much shorter prognostic period (1 y), players (*n* = 353), from across several age groups (U9-U21), retained by an English academy at the end of the season were generally taller and heavier than those released (Patel et al., 2020). Thus, it is possible that in England, in the short-term, stature, and body mass might perhaps influence who is released or retained in an academy, and perhaps encourage the development of a playing population which is selected on this basis. However, the findings from the present study suggest that, over the longer-term, these anthropometric characteristics do not influence who achieves professional playing status at a senior level. Also, the random effects outputs from the modeling undertaken in this study suggest there is considerable inter-individual variation in stature and body mass among all the players studied, and this adds support to the conclusion that stature and body mass are not key influences on attainment of professional playing status.

Many papers exclude goalkeepers, partly because they are seen to be a very different positional group from all other outfield players and also because often in any sample there are relatively few goalkeepers (White et al., 2018). Given the sample size in the current study, it was possible to examine differences between positional groups including goalkeepers. Throughout their development the goalkeepers were taller and heavier than all other positional groups and their performance was poorer in all four of the field-based tests (at age 13.0 the models predict the goalkeepers were 3.4–3.6 cm taller, 4.1–4.4 kg heavier, 0.17–0.24 m.s^−1^ slower in the 20-m sprint test, 0.13–0.17 m.s^−1^ slower in the slalom agility test, jumped 0.9–1.6 cm less in the vertical counter-movement jump with arm swing test, and ran 235–305 m less in the multistage fitness test). Therefore, these findings confirm that, compared to other positional categories, goalkeepers are anthropometrically larger and have inferior performance, although it should be recognized that the tests used in the current study are more orientated toward outfield player performance (White et al., 2018). Interestingly, forwards were faster than all other positions in the 20-m sprint and slalom agility tests (at age 13.0 by 0.06–0.24 m.s^−1^ and 0.04–0.17 m.s^−1^, respectively) and they also jumped higher than all other positions (at age 13.0 by 0.8–1.7 cm). While some other differences did exist between the outfield playing positions, generally outfield players were much more similar to each other than they were to goalkeepers, which is in-line with previous cross-sectional analyses in elite youth football players (Deprez et al., 2015a). However, regardless of any differences that might be evident between positional groups, within players in a particular position the professional players outperformed their non-professional peers.

The observation that those players who achieved professional status had better performance (and by implication better physical capabilities) than their non-professional peers in speed, slalom agility, vertical jumping and endurance tasks, and also had different longitudinal patterns of development for slalom agility and vertical counter-movement jump with arm swing test performance, emphasizes the importance of performance testing and underlines its utility in the talent identification and development process. Despite the clearly better performance in the future professional players viewed retrospectively, a typical scenario faced by practitioners from a talent identification and development perspective is the necessity to make judgements about a player's capabilities in the period when the measurements are being made. It is an interesting question how useful the tests examined in the current study are for that purpose, especially given the time, effort and resources expended, and the (perhaps) inherent assumption that talent identification and development is the primary reason for such data collections, and the directives contained in documents such as the EPPP. These types of debate have been had previously (Mendez-Villanueva and Buchheit, 2013). Because the current study allows the variation in physical performance across individuals to be quantified, it shows that the differences in physical performance between the non-professional and professional groups is smaller than the inter-individual variation across all the players (the difference in performance across the four field-based tests between the non-professional and professional playing groups represents 6–7% of the inter-individual variation in 20-m sprint and multistage fitness test performance in all players, and up to 11 and 21% (at age 18.0) for the vertical counter-movement jump with arm swing test and slalom agility test, respectively). Consequently, at the time of measurement it could be argued that it is unlikely that performance on field-based tests will allow an individual future professional player to be distinguished and therefore perhaps the time, effort and resources involved in such performance field testing has some limits, certainly from a talent identification and development perspective. This observation also highlights perhaps the dangers of using one-off measurements of physical performance as a basis for early (de)selection of players (Vaeyens et al., 2008). But the present study clearly demonstrates that the professional players were better physical performers when the non-professional and professional groups were compared. Indeed, the magnitude of some the differences between the groups (based on effect size) was moderate (*d* = 0.5 at 18.0 y for slalom agility) and certainly at older ages large enough to be measurable. Also, it could be argued that even small magnitude differences in performance (*d* = 0.2 at 13.0 and 18.0 y for 20-m sprint speed) could be crucial at the elite level.

Furthermore, time spent conducting field-based tests ensures performance development is monitored, and maintained and perhaps even improved. It allows the identification of strengths and weaknesses in individuals and the effectiveness of training programmes to be accurately evaluated (Svensson and Drust, 2005). Also, such testing has value in evaluating a player recovering from injury. Thus, an ongoing programme of physical field “testing” helps provide players with the most appropriate learning environment to realize their potential. That is why, even if one thinks the physical tests examined in this study may have some limits in a practical context when trying to distinguish between future professional and non-professional players at the time of measurement, it is likely that time spent longitudinally monitoring and testing physical performance is worthwhile within elite talent development programmes in football. The current analysis allows practitioners to assess their players' longitudinal development of physical characteristics across exact chronological age in relation to academy players of varying positions who eventually turned professional, while also being able to consider the normal range of performance at each age and the normal range of rate of development, for each physical characteristic. Thus, the present findings could be used to support a long-term field-based testing programme for the identification of potential strengths and weaknesses in individuals across time, and for evaluating the effectiveness of individualized training plans.

The present study supports the notion that the development of physical characteristics (such as speed, agility, vertical jumping, and endurance), is dynamic, variable, and non-linear (Simonton, 1999; Abbott et al., 2005), and that talent identification and development should be viewed as a long-term process (Sarmento et al., 2018). Consequently, the study's findings encourage understanding the general patterns of development of stature, body mass, speed, agility, vertical jumping, and endurance in growing football players, as well-understanding the “normal” deviations from these general patterns, allowing a more individualized approach to supporting players' physical development, and taking a more long-term approach toward developing speed, agility, vertical jumping, and endurance in players.

Although this study is the first to follow the physical development of a large sample of young male football players from the English talent development system through to professional status, there are some potential limitations with the analysis that is presented here. During adolescence in particular, the variation in the speed of progression toward the adult state means that chronological age may not be a particularly good indicator of maturational status. In males, most of the changes associated with the adolescent growth spurt (such as increases in muscle mass and strength) are likely to have positive effects on performance (Philippaerts et al., 2006). Hence, variation in maturational status between the players investigated in the study that is not accounted for by chronological age could have influenced the results presented here. Given that the players were drawn from 16 different academies it is likely that the training and match environments to which they were exposed will have varied (in terms of type, intensity, duration), and obviously this variation could have influenced the findings of the present study. Furthermore, as seniors, this sample of players made appearances across 65 professional leagues across the world. While this may offer a more inclusive and comprehensive view of professional success compared to previous studies which have tended to judge future success based on appearances in just one country [e.g., Leyhr et al. (2018) considered appearances in German leagues only], it is likely there was considerable variation in the characteristics and standard of play in the 65 leagues considered, and this could have influenced the current results. While beyond the scope of the current study, future research may examine the development of academy footballers in relation to success in particular leagues and countries. Finally, this paper concerns itself only with anthropometric and physical performance characteristics. These are important, but clearly there are a number of other factors which contribute to the development of, and the successful progression of the academy football player to professional status (such as technical ability and game sense, psychological skills, family background), and the influence of these was not considered in this study (Huijgen et al., 2014).

## Conclusion

In summary, of the 2,875 players examined in the current study, which covered the full 10 year duration of a football talent development programme in England, 23% went on to play at least one professional game, and while these players were not taller or heavier than their non-professional peers, from 12 y the future professionals produced better slalom agility and vertical counter-movement jump with arm swing test performance than future non-professionals, and the longitudinal pattern of development of these characteristics improved at a faster rate in the future professional players. Future professional players were also faster over 20-m and ran further in the multistage fitness test than future non-professional players throughout development. Whereas, multistage fitness test performance improved linearly with age, the development of all other physical characteristics was non-linear. There were inter-individual differences in the development of all characteristics, and there were differences between playing positions in the development of all characteristics. Thus, in summary, future professional players are more agile, better vertical jumpers, faster and more endurance fit than future non-professional players as they age, and the pattern of development is different in professional players and non-professional players for slalom agility and vertical jumping performance.

## Data Availability Statement

The datasets presented in this article are not readily available because, although the names of participants were removed before data were analyzed, the data itself contains information that could be identifiable given the availability of publicly available information (e.g., squad members, staff members, dates), the year of the season, and the identification of the clubs involved (by deducing from the author affiliations). Furthermore, participants agreed to have their data used in the current study, but not to have their data shared with external parties, which presents an ethical challenge. Requests to access the datasets should be directed to Chris Saward, chris.saward@ntu.ac.uk.

## Ethics Statement

The studies involving human participants were reviewed and approved by The Ethical Advisory Committee for Research Involving Human Participants, Loughborough University and the Human Invasive Ethical Review Committee, College of Science and Technology, Nottingham Trent University. Written informed consent to participate in this study was provided by the participants' legal guardian/next of kin.

## Author Contributions

MN, CSu, JM, MH, HG, and CSa contributed to the conception and design of the study. CSa, MH, JM, and HG performed the data collection. CSa and JM performed the data analysis. CSa, JM, and MN wrote the first draft of the paper. All authors contributed to manuscript revision and read and approved the submitted version.

## Conflict of Interest

MH is employed by Manchester United Football Club. The remaining authors declare that the research was conducted in the absence of any commercial or financial relationships that could be construed as a potential conflict of interest.
