# Supplementary figures and images for: Longitudinal Physical Development of Future Professional Male Soccer Players: Implications for Talent Identification and Development?
Source: Front Sports Act Living. 2020 Oct 21;2:578203. doi: 10.3389/fspor.2020.578203 (PMC7739714; doi:10.3389/fspor.2020.578203)

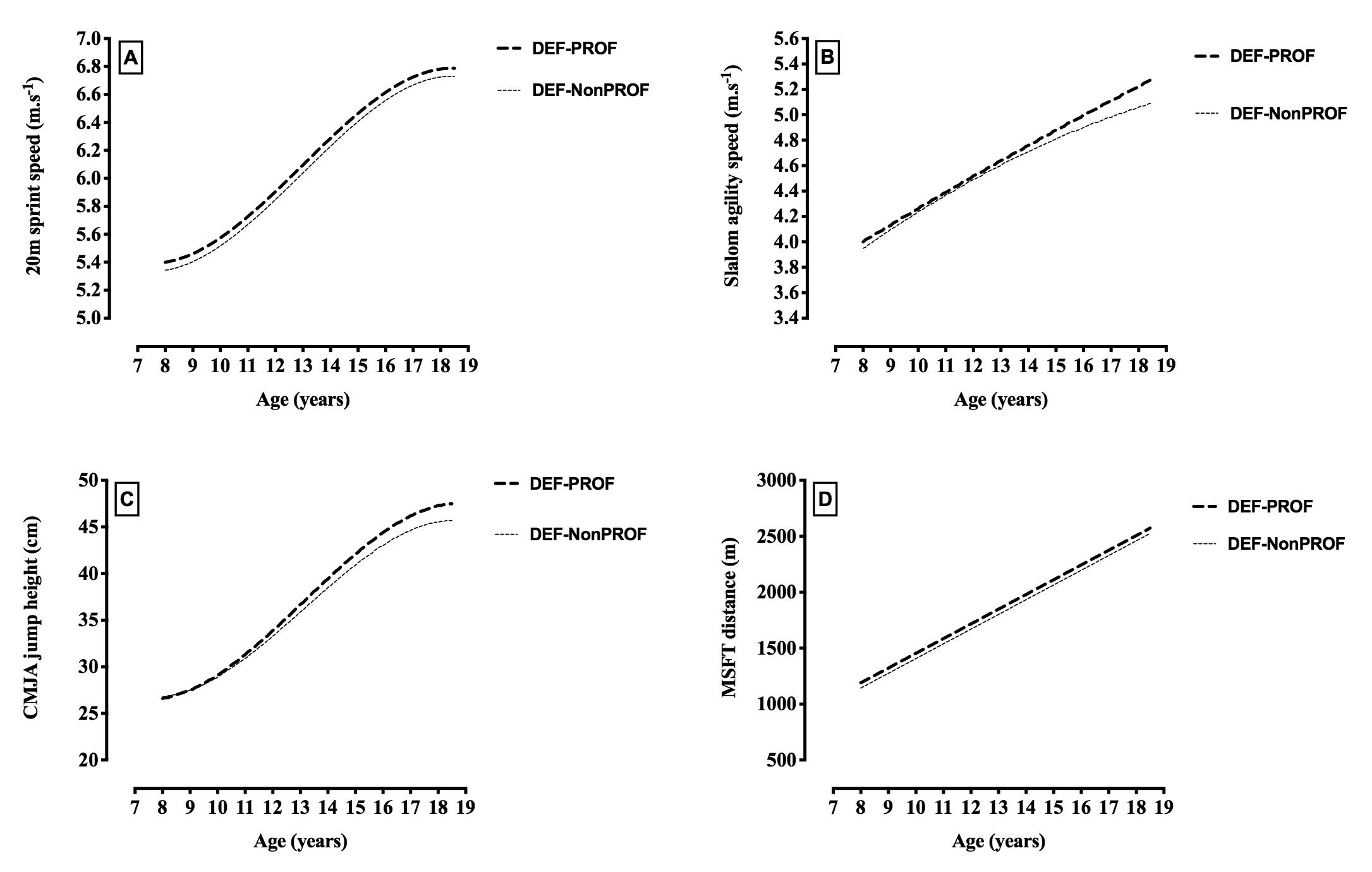

Supplement: Supplementary file 1 [file Image_1.JPEG]

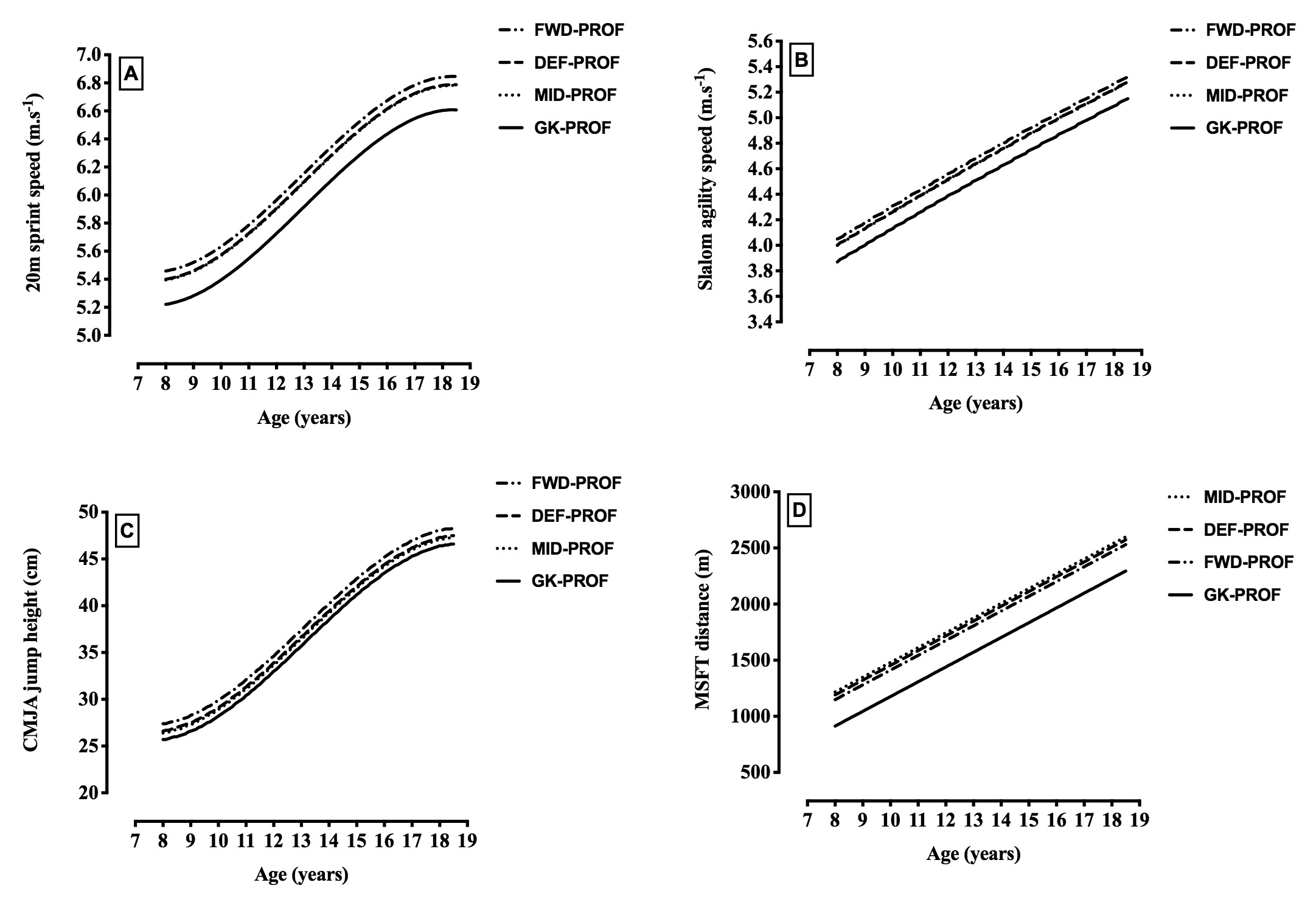

Supplement: Supplementary file 2 [file Image_2.JPEG]

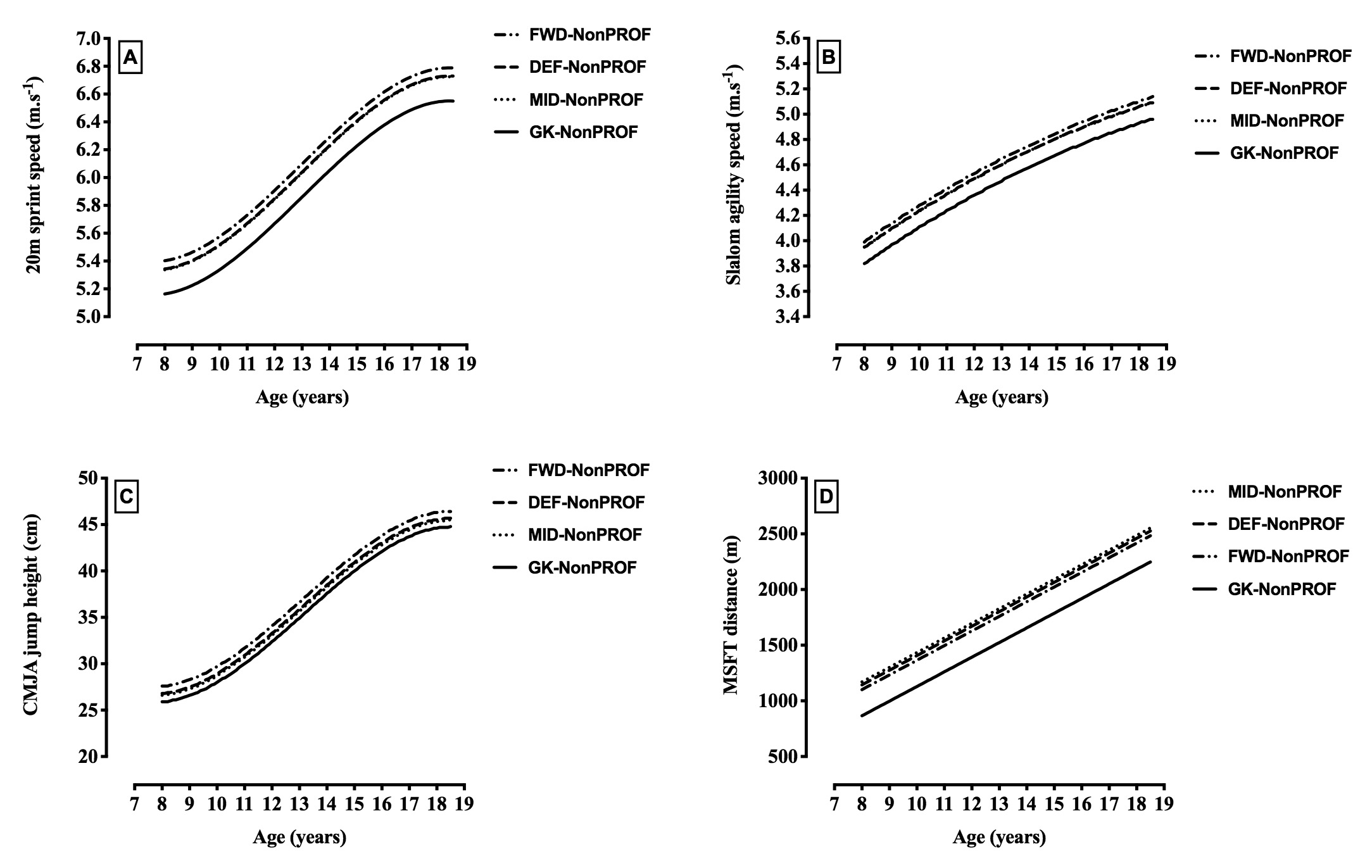

Supplement: Supplementary file 3 [file Image_3.JPEG]
