# Supplementary material for: Longitudinal Physical Development of Future Professional Male Soccer Players: Implications for Talent Identification and Development?
Source: Front Sports Act Living. 2020 Oct 21;2:578203. doi: 10.3389/fspor.2020.578203 (PMC7739714; doi:10.3389/fspor.2020.578203)
Supplement: Supplementary file 4 [file Table_1.DOCX]

These supplemental figures were requested by one of the reviewers of the paper.

Figure 7. Changes in: A) 20-m sprint speed (m.s^-1^); B) slalom agility speed (m.s^-1^); C) vertical counter-movement jump with arm swing (CMJA) height (cm); and D) multistage fitness test (MSFT) distance (m) with age by future playing standard (PROF = players who made at least one professional league appearance; NonPROF = players who DID NOT make any professional league appearances). The data is based on the defender position and on the fixed parameter estimates from the models described in Table 3.

Figure 8. Changes in: A) 20-m sprint speed (m.s^-1^); B) slalom agility speed (m.s^-1^); C) vertical counter-movement jump with arm swing (CMJA) height (cm); and D) multistage fitness test (MSFT) distance (m) with age by playing position (PROF = players who made at least one professional league appearance). Data is based on the fixed parameter estimates from the models described in Table 3.

Figure 9. Changes in: A) 20-m sprint speed (m.s^-1^); B) slalom agility speed (m.s^-1^); C) vertical counter-movement jump with arm swing (CMJA) height (cm); and D) multistage fitness test (MSFT) distance (m) with age by playing position (NonPROF = players who DID NOT make any professional league appearances). Data is based on the fixed parameter estimates from the models described in Table 3.
